# Digital self‐monitoring: Does adherence or association with outcomes differ by self‐monitoring target?

**DOI:** 10.1002/osp4.391

**Published:** 2019-12-12

**Authors:** Meghan L. Butryn, Kathryn M. Godfrey, Mary K. Martinelli, Savannah R. Roberts, Evan M. Forman, Fengqing Zhang

**Affiliations:** ^1^ Department of Psychology Drexel University Philadelphia PA USA

**Keywords:** monitoring, obesity treatment, weight loss

## Abstract

**Objective:**

Digital self‐monitoring of eating, physical activity, and weight is increasingly prescribed in behavioural weight loss programmes. This study determined if adherence rates or associations with outcomes differed according to self‐monitoring target (ie, self‐monitoring of eating versus physical activity versus weight).

**Methods:**

Participants in a 3‐month, group‐based weight loss programme were instructed to use an app to record food intake, wear a physical activity sensor, and use a wireless body weight scale. At post‐treatment, weight loss was measured in clinic and moderate‐to‐vigorous physical activity (MVPA) was measured by research‐grade accelerometer.

**Results:**

Adherence to self‐monitoring decreased significantly over time for eating and weight but not physical activity. Overall, adherence to self‐monitoring of weight was lower than that of eating or physical activity. Greater adherence to self‐monitoring of eating, physical activity, and weight each predicted greater weight loss. Only greater adherence to self‐monitoring of eating was associated with greater bouted minutes of MVPA.

**Conclusions:**

Findings from this study suggest that self‐monitoring should be considered a target‐specific behaviour rather than a unitary construct when conceptualizing adherence and association with treatment outcomes.

## 1 INTRODUCTION

Self‐monitoring is a cornerstone of the behavioural approach to weight loss.^1^ Participants in behavioural weight loss programmes are generally instructed to track eating behaviour, physical activity, and weight. Self‐monitoring is thought to promote behaviour change (and ultimately weight loss) by helping participants to regularly assess progress towards goals and compliance with programme recommendations, increase awareness of key behavioural patterns, alert participants to areas of concern, and provide a sense of accomplishment for successes.^2^ Traditionally, self‐monitoring has been conducted in analogue forms, by using a paper diary to record food intake and minutes of physical activity and weighing oneself with a conventional bathroom scale. Increasingly, behavioural weight loss participants have access to digital tools: a smartphone app to record food intake, a wearable physical activity sensor, and a wireless body weight scale that syncs to an app.

Although research on digital self‐monitoring is growing quickly, more clarity is needed regarding the extent to which self‐monitoring should be treated as a unitary construct or if there are important differences in self‐monitoring process and outcomes depending on the self‐monitoring target (ie, eating versus physical activity versus weight). The amount of effort or time required to enter and review the data, the extent to which the information provided may be perceived as threatening or upsetting, or the perceived value of the data may differ depending on the self‐monitoring target. Research is needed that compares digital self‐monitoring of eating, physical activity, and weight to determine if they differ in adherence rates, changes in adherence over time, or associations with objectively measured treatment outcomes (namely, weight loss and adoption of physical activity).

The present study is one of few that prescribed and evaluated use of a comprehensive suite of digital self‐monitoring tools in an in‐person, group‐based behavioural weight loss programme and compared self‐monitoring of eating, physical activity, and weight. Aim 1 was to test the hypothesis that adherence rates would differ by target of self‐monitoring. Specifically, self‐monitoring of physical activity was hypothesized to be higher than that of weight and eating, given that the method for physical activity self‐monitoring in this study required minimal effort and that information about physical activity may be perceived as less threatening to participants than information about weight or calorie intake. Similarly, Aim 2 was designed to evaluate the hypothesis that decline in self‐monitoring adherence over time would be lowest for physical activity compared with eating and weight self‐monitoring. Aim 3 was to test the hypothesis that greater adherence to each type of self‐monitoring would be associated with better weight and physical activity outcomes. Monitoring progress towards goals (whether successful in meeting those goals or not) could be expected to increase motivation or self‐efficacy for multiple weight control behaviours (eg, tracking weight loss progress and being pleased with progress could promote physical activity adherence, if a participant believes that physical activity is contributing to success; tracking calorie intake and being disappointed with progress could also promote physical activity adherence, if a participant believes adherence to physical activity may be necessary to compensate for high calorie intake).

## 2 METHODS

### 2.1 Participants

This study was part of a larger parent project (Clinical Trials Identifier: NCT03337139) that was designed to evaluate the feasibility and efficacy of an experimental weight loss maintenance intervention. In the parent study, all participants received the same behavioural, face‐to‐face, group‐based treatment during the weight loss phase of the study (weeks 1‐12). At the end of that phase, participants were randomized to two different weight loss maintenance conditions (weeks 13‐52). During the weight loss maintenance phase, all intervention contact occurred via weekly text messages and monthly phone calls, and the two conditions varied according to the access that coaches had to participant self‐monitoring device data. The current substudy was focused on outcomes for all participants enrolled in the uniform weight loss phase of treatment (weeks 1‐12).

Participants were 18 to 70 years old, had a BMI of 25 to 45 kg/m^2^, had a smartphone and home wireless access, and had no contraindications to physical activity. Participants were ineligible if they had medical or psychiatric conditions that may have posed a risk during lifestyle modification, had recently began or changed the dose of a medication that may affect weight loss, or had a weight loss of greater than or equal to 5% in the previous 3 months. Women who were currently nursing, pregnant, or planning to become pregnant over the course of the study were also ineligible. All participants provided written informed consent upon enrolment. Participants were not compensated for assessments within the 12 weeks of weight loss treatment. All study procedures were approved by the Institutional Review Board of Drexel University.

### 2.2 Intervention

Participants were provided with one treatment orientation session (week 0) followed by 12 weekly behavioural weight loss group sessions, delivered face‐to‐face in groups of approximately 12 participants. The treatment protocol was adapted from Look AHEAD and the Diabetes Prevention Program.^3^, ^4^ It included strategies for stimulus control, problem solving, and goal setting. A physical activity progression was prescribed for increasing days and minutes of moderate‐to‐vigorous physical activity, ultimately reaching a goal of 250 bouted minutes per week by 12 weeks.

Participants were provided with digital self‐monitoring tools for eating, physical activity, and weight and instructed to use them for the duration of treatment. Participants installed the Fitbit app on their smartphones and were asked to record all food and beverage intake. The app allowed participants to look up items in a comprehensive database, add custom foods and meals to their food logs, and add foods by scanning barcodes. The importance of recording intake as soon as possible after eating was emphasized. Participants were instructed to use their logs to gauge progress for meeting calorie goals.

Participants also were provided with the Fitbit Flex, a physical activity sensor worn on the wrist that has established reliability and validity.^5^, ^6^ Participants could view physical activity data in the Fitbit app and were instructed to track “active minutes” (ie, bouts of moderate‐to‐vigorous physical activity) in order to track progress in meeting physical activity goals. Participants were told that they could remove the device at night, but should wear it at all other times.

A Yunmai Smart Scale was provided for self‐monitoring of weight, which also synced with the Fitbit app. Participants were instructed to weigh themselves weekly in weeks 1 to 10 and daily in weeks 11 and 12. (Participants were told that programme advised against self‐monitoring weight more frequently than daily; otherwise, weighing more frequently than weekly during weeks 1 to 10 was neither encouraged nor discouraged.) This prescription was designed to align with the traditional approach of self‐monitoring weight weekly during active weight loss and daily during weight loss maintenance^7^; participants were expected to be preparing for weight loss maintenance as the end of in‐person treatment approached. Participants were told that daily weighing after 10 weeks was advised because (a) it would provide accountability that otherwise would be decreasing when the initial phase of weight loss treatment ended at 12 weeks and (2) during the weight loss maintenance phase of treatment, risk for relapse would heighten, and daily weighing could reduce that risk by identifying small weight gains early.

### 2.3 Measures

#### 2.3.1 Demographics

A self‐report questionnaire was used to record participant's age, gender, race, and ethnicity at baseline.

#### 2.3.2 Height and weight

Project staff used a Tanita model WB‐3000 digital scale to weigh participants (weighed in street clothes without shoes) at baseline and at week 12. Height was measured at baseline using a mechanical height rod equipped on the scale.

#### 2.3.3 Physical activity

ActiGraph GT3X tri‐axial, solid‐state accelerometers were used to measure minutes of moderate‐to‐vigorous physical activity (MVPA) at the end of treatment. Actigraph data were not collected at baseline in order to minimize participant burden and because randomization for the parent study did not occur until 12 weeks (ie, the beginning of the weight loss maintenance phase). A minimum of four days of valid accelerometer data was required for inclusion in analyses. ActiLife software was used to calculate participant's MVPA using established cut‐points.^8^ Bouted and unbouted MVPA were each examined. This programme's prescription for physical activity was given in bouted minutes (ie, exercise completed in episodes of at least 10 min each), which is also what Fitbit measured for active minute calculations, but the newest guidance indicates unbouted physical activity is a more appropriate intervention target.^9^

#### 2.3.4 Self‐monitoring adherence

Adherence to self‐monitoring was assessed with a research portal that remotely and automatically captured data from participants' devices. Participants consented to sharing these data with the research team. The intervention team did not have access to the data during weight loss treatment. (Participants printed their food records in order to receive feedback on their dietary intake.) For dietary self‐monitoring, percent adherence was determined by the days in which there was at least one item logged (consistent with the approach used in other studies).^10^ For physical activity self‐monitoring, percent adherence was determined by the number days in which participants had at least 500 steps tracked on the Fitbit.^11^, ^12^, ^13^ Percent adherence to weight self‐monitoring was calculated based on adherence to the varied self‐weighing prescription: once per week from weeks 1 to 10 and once per day for weeks 11 to 12. When weight self‐monitoring was examined as a predictor of 12‐week outcomes, only adherence during weeks 1 to 10 was included, so that the greater number of data points closest in time to outcomes measurement would not bias the predictor variable.

### 2.4 Data analysis

Repeated measures analysis of variance (ANOVA) analyses were used to examine change in self‐monitoring over time and compare self‐monitoring adherence across targets. For distributions that violated the assumption of normality, non‐parametric statistical tests (avoperm)^14^ were run to produce permutation‐based *t* and *P* values. Multiple regressions were used to examine associations between self‐monitoring adherence and outcomes including weight loss and unbouted MVPA. As the distribution of bouted MVPA was zero‐inflated, compound Poisson linear regression models (cplm)^15^ were used. All analyses were conducted in R.^16^

## 3 RESULTS

### 3.1 Preliminary analyses

There were 87 participants enrolled in the parent study (M_age_ = 50.0 ± 13.1 y, M_BMI_ = 34.9 ± 4.9 kg/m^2^). The sample was composed primarily of participants identifying as White (51.7%) or Black/African American (35.6%), and 82.8% of participants were female. Complete demographic information is available in Table 1. Participants attended an average of 82% of group sessions. Data were missing for 10 participants who discontinued their participation during the 12‐week treatment (88.5% of the sample retained), and thus, analyses for the current study are based on 77 participants.

**Table 1:** Baseline characteristics

	Mean (SD) or frequency (%)
Age (years), *M* (SD*)*	50.02 (13.14)
Ethnicity, n %	
Hispanic/Latino	5 (5.7%)
Non‐Hispanic	82 (94.3%)
Race, n %	
American Indian/Alaskan Native	1 (1.1%)
Asian	3 (3.4%)
Native Hawaiian or other Pacific Islander	0 (0%)
Black or African American	31 (35.6%)
White	45 (51.7%)
Other or more than once race	7 (8.0%)
Education, n %	
High school or lower	6 (6.9%)
Associate's degree	16 (18.4%)
Bachelor's degree	27 (31.0%)
Graduate or professional degree	38 (43.7%)
Household income, n %	
$0‐$25 000	7 (8.1%)
$25 000‐$50 000	14 (16.3%)
$50 000‐$75 000	18 (20.9%)
$75 000‐$100 000	20 (23.3%)
$100 000‐$125 000	7 (8.1%)
$125 000 or higher	20 (24.3%)
Marital status, n %	
Married	39 (44.8%)
Widowed	2 (2.3%)
Divorced	15 (17.2%)
Separated	3 (3.4%)
Never married	28 (32.2%)
BMI, M (SD)	34.9 (4.9)
Weight (kg), M (SD)	94.5 (14.5)

### 3.2 Self‐monitoring adherence by target

Mean self‐monitoring adherence was 91.5% (SD = 12.6%) for eating, 93.6% (SD = 13.9%) for physical activity, and 85.6% (SD = 18.7%) for weight. Physical activity and dietary self‐monitoring adherence were not significantly different across the 12 weeks (*F*_1,76_ = 3.15, *P* = .08). Weight self‐monitoring adherence was less than physical activity (*F*_1,76_ = 28.21, *P* < .001) and dietary (*F*_1,76_ = 17.5, *P* < .001) self‐monitoring adherence.

### 3.3 Change over time

**Figure 1:**
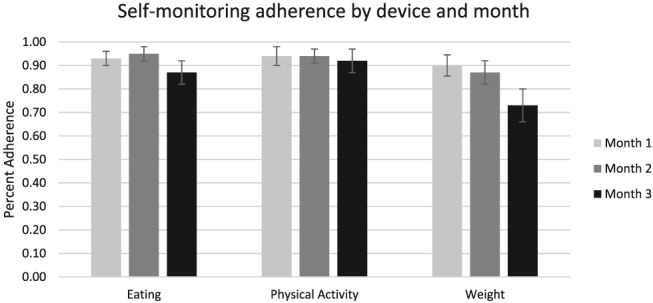
Self‐monitoring adherence by device and month

Change over time in self‐monitoring adherence is shown in Figure 1. Results of non‐parametric repeated measures ANOVA indicated that adherence to self‐monitoring of eating changed significantly over time (Month 1 = 92.7%, Month 2 = 94.9%, Month 3 = 86.7%, *F*_2,152_ = 7.49, permutation‐based *P* < .001). Self‐monitoring adherence did not change significantly over time for physical activity (Month 1 = 94.5%, Month 2 = 94.0%, Month 3 = 92.4%, *F*_2,152_ = 0.75, permutation‐based *P* = .48).

Participants were instructed to self‐monitor their weight weekly until the final 2 weeks of treatment, at which time daily self‐monitoring of weight was to begin. An average of 89.6% (SD = 21.6%) and 87.3% (SD = 22.1%) of participants each week engaged in weight self‐monitoring during Months 1 and 2, respectively. The average frequency of weight self‐monitoring was 2.0 days per week during Month 1 and 2.0 days per week during Month 2. Adherence did not significantly differ from Month 1 to Month 2 (*F*_1,76_ = 0.85, permutation‐based *P* = .37). During the final 2 weeks of treatment, when weight self‐monitoring was to be conducted daily, this behaviour was observed on an average of 72.8% of days (SD = 30.3%). Adherence during the final 2 weeks was significantly lower than that during Month 1 (*F*_1,76_ = 26.79, *P* < .001) and Month 2 (*F*_1,76_ = 17.75, permutation‐based *P* < .001).

Given that changes in adherence were observed over time, adherence by target of self‐monitoring was compared separately during each of the 3 months of treatment. Physical activity self‐monitoring adherence was higher than dietary self‐monitoring adherence during Month 1 (*F*_1,76_ = 5.03, *P* = .03) and Month 3 (*F*_1,76_ = 5.15, *P* = .03). There was no significant difference during Month 2 (*F*_1,76_ = 0.91, *P* = .34).

Physical activity self‐monitoring adherence was higher than weight self‐monitoring adherence during Month 1 (*F*_1,76_ = 6.31, *P* = .01) and Month 2 (*F*_1,76_ = 12.27, *P* < .001) and during the period in Month 3 when daily self‐monitoring of weight was prescribed (*F*_1,76_ = 38.71, *P* < .001).

Dietary and weight self‐monitoring adherence were not significantly different during Month 1 (*F*_1,76_ = 2.81, *P* = .10). Dietary self‐monitoring adherence was greater than weight self‐monitoring adherence during Month 2 (*F*_1,76_ = 13.64, *P* < .001) and during the period in Month 3 when daily self‐monitoring of weight was prescribed (*F*_1,76_ = 25.03, *P* < .001).

### 3.4 Association with outcomes

At 12 weeks, participants had lost an average of 6.1% of initial weight and were engaging in 152.0 minutes per week of bouted MVPA and 334.4 minutes per week of unbouted MVPA (as measured by Actigraph accelerometers). Self‐monitoring adherence was examined as a predictor of 12‐week outcomes controlling for race, age, gender, and baseline BMI. Greater adherence to self‐monitoring of eating (*t*_57_ = 4.47, *P* < .001), physical activity (*t*_57_ = 3.23, *P* = .002), and weight (*t*_57_ = 3.60, *P* < .001) each predicted greater weight loss. Only greater adherence to self‐monitoring of eating was associated with greater bouted minutes of MVPA (*t*_52_ = 2.12, *P* = .04). Greater adherence to self‐monitoring of eating (*t*_52_ = 3.88, *P* < .001) and physical activity (*t*_52_ = 2.82, *P* = .007) was each associated with greater unbouted minutes of MVPA.

**Figure 2:**
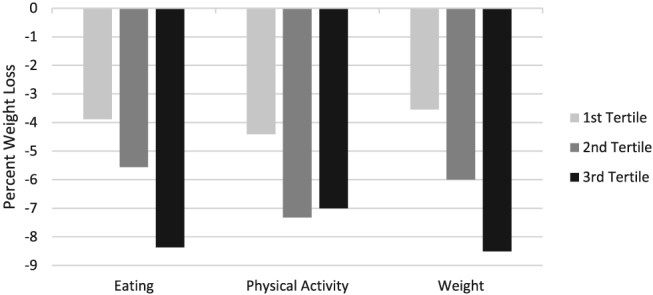
Percent weight loss from baseline to 12 weeks by self‐monitoring adherence

**Figure 3:**
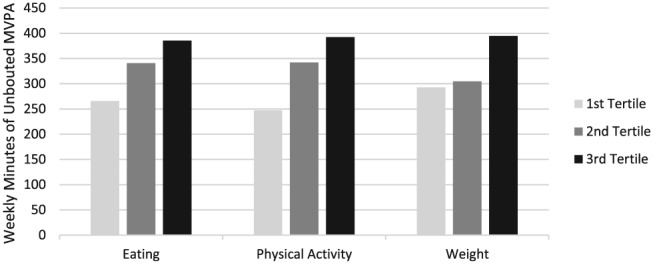
MVPA (unbouted) minutes by self‐monitoring adherence

To illustrate the clinical significance of these relationships, participants were divided into tertiles of self‐monitoring adherence. Figure 2 shows the relationship between weight loss and self‐monitoring adherence in each target. Figure 3 shows the relationship between unbouted physical activity minutes and self‐monitoring adherence in each target.

## 4 DISCUSSION

In the current study, participants were provided with a 12‐week, group‐based, behavioural weight loss intervention and instructed to engage in digital self‐monitoring of eating, physical activity, and weight. There were meaningful differences in total adherence, change in adherence over time, and association with outcomes depending on the target of self‐monitoring, which suggests that in clinical work and future research, self‐monitoring should be considered as a target‐specific behaviour, rather than a unitary construct.

Overall, adherence to self‐monitoring was high, and engagement remained impressive throughout the treatment period. On over 90% of days during the 12‐week treatment period, participants recorded at least one item of food/beverage consumed in their app and wore their physical activity sensor. However, a significant reduction in adherence to self‐monitoring of eating was observed over time, which is consistent with previous research.^17^, ^18^ Logging food and beverage intake in an app can be effortful and time consuming, taking an average of 15 to 20 minutes per day,^19^ and behavioural fatigue may result. It is possible that the perceived value of dietary self‐monitoring decreased as treatment progressed because participants had already learned a substantial amount about nutrition or about their eating patterns. There is some evidence that adherence to dietary prescriptions is greatest in the early weeks of weight loss treatment^20^; if participants began having more dietary lapses later in treatment, they may have been more likely to avoid self‐monitoring of eating as a result.

No change over time in adherence was observed for physical activity self‐monitoring, suggesting that this form of self‐monitoring may be easiest to maintain (in terms of effort or time required), most valued by participants, or more positive in terms of the affective response to the data collected. Adherence was greater for physical activity self‐monitoring than eating self‐monitoring during Months 1 and 3 (there was no difference during Month 2), which further suggests that the user experience for these two forms of self‐monitoring may differ in important ways that should be studied further. Two previous studies found that self‐monitoring of physical activity significantly decreased over time,^21^, ^22^ but both of those studies used analogue forms of self‐monitoring, rather than a digital sensor. Self‐monitoring with a digital sensor presumably requires less effort than recording bouts of physical activity in a paper log and thus that may lead to greater maintenance of physical activity self‐monitoring than has been observed in previous studies. When weekly self‐monitoring of weight was prescribed, close to 90% adherence was observed. In fact, on average, participants weighed themselves twice per week during this period. The lowest rates of adherence were observed for daily self‐monitoring of weight (prescribed during weeks 11 and 12 of treatment), which occurred on 73% of days during the prescribed period. Overall, adherence to self‐monitoring of weight was lower than that of eating or physical activity. There may be unique challenges for self‐monitoring of weight that need to be addressed clinically, such as a sense of shame regarding one's weight (regardless of recent progress) or avoidance that results when one fears that recent weight change will be disappointing.^23^ However, it is difficult to interpret the decline in adherence to weight self‐monitoring because the prescription for frequency of weight self‐monitoring changed from weekly to daily during the final weeks of the programme, and thus, the change in frequency prescribed is confounded with the timing of the initial weight loss phase of treatment coming to an end.

Each type of self‐monitoring (eating, physical activity, and weight) was associated with weight loss. Previous research has also documented a link between weight loss and self‐monitoring of diet,^10^, ^18^, ^19^, ^24^ self‐monitoring of weight,^24^, ^25^, ^26^ and self‐monitoring of physical activity.^21^, ^27^ It remains unclear for each type of self‐monitoring how much the link between adherence and weight loss can be explained by underlying level of motivation or self‐regulation ability. Experimental research that has manipulated the provision or prescription of digital self‐monitoring tools and examined the resulting effect on weight loss has yielded mixed results.^28^, ^29^, ^30^, ^31^, ^32^, ^33^ Additional experimental research is needed to determine the extent to which each type of digital self‐monitoring directly improves weight loss and, if there is a causal relationship, identify the specific mechanism of action.

Participants who had the greatest adherence to dietary self‐monitoring also had the highest levels of bouted and unbouted physical activity at 12 weeks. Adherence to physical activity self‐monitoring was associated with unbouted, but not bouted, physical activity outcomes. Adherence to weight self‐monitoring was not associated with physical activity at 12 weeks. Few studies have examined the relationship between dietary self‐monitoring and physical activity outcomes. Reed and colleagues^34^ compared participants with high versus low adherence to dietary self‐monitoring on a mobile tracking app and found that the two groups did not significantly differ on self‐reported physical activity outcomes following a 12‐week community weight loss programme. One explanation for the differences in findings between the current study and prior research is that Reed et al^34^ relied on a self‐report, retrospective measure of physical activity outcome, whereas the current study used objective, real‐time measurement of physical activity. The current study found that greater adherence to dietary self‐monitoring is associated with greater unbouted and bouted physical activity at 12 weeks. Previous research has demonstrated a “spill‐over” effect whereby general treatment motivation and exercise‐specific motivation contribute to improved eating self‐regulation.^35^ Thus, participants with higher adherence to dietary self‐monitoring in this study may have been more motivated regarding general and/or exercise‐specific adherence.

Previous research has documented a relationship between physical activity self‐monitoring and physical activity outcomes,^21^, ^27^ however, these studies relied on self‐report measures of physical activity outcomes and did not distinguish between bouted and unbouted physical activity. Previous studies also relied on manual forms of self‐monitoring (ie, by asking participants to record minutes of activity into paper or digital diaries), whereas the present study defined self‐monitoring of physical activity by the number of days participants wore their Fitbit. It is unclear why self‐monitoring of physical activity was associated with unbouted, but not bouted, physical activity. One possible explanation is that, despite being told to track progress using number of active minutes on their Fitbit, participants may have used other Fitbit® metrics such as daily steps to track progress, which may correspond more to unbouted, rather than bouted, activity levels. Alternatively, given findings from Carels,^27^ which showed that greater self‐monitoring of physical activity via paper diaries was associated with higher weekly self‐reported minutes of planned exercise but not self‐report leisure time activity, it is possible that analogue forms of self‐monitoring influence changes in bouted physical activity whereas passive activity sensors influence change in lifestyle activity. Future research should assess whether using an activity tracker is more likely to influence changes in lifestyle activities, bouted exercise, and/or overall levels of physical activity. Adherence to weight self‐monitoring was not associated with physical activity at 12 weeks in the current study. This finding is consistent with prior cross‐sectional research in normal weight and overweight young adults, which found that frequency of self‐weighing was not associated with objectively measured physical activity.^36^ The findings from the current study are not consistent with previous research, which demonstrated a significant relationship between self‐weighing and daily steps, measured objectively using a accelerometry,^37^, ^38^ or activity measured via self‐report.^37^ Of note, these studies differed in their prescriptions for self‐monitoring of weight, instructing participants to weigh daily^38^ or every other day,^37^ whereas the current study relied on weekly self‐weighing for weeks 1 to 10 and daily weighing for weeks 11 to 12. Future research is needed to clarify the relationship between self‐weighing frequency and self‐monitoring adherence with physical activity outcomes.

The key strengths of this study are the objective measurement of self‐monitoring adherence and weight change and use of research‐grade accelerometer to measure physical activity. Both unbouted and bouted measures of physical activity were evaluated, allowing for interpretation of results based on the current recommendations for physical activity.^9^ The sample was racially diverse and retention at 12 weeks was good. Examining adherence and outcomes for each of three types of digital self‐monitoring that are increasingly prescribed in behavioural weight loss allows this study to make a unique contribution to the literature.

The study also had notable limitations. The sample was composed primarily of female participants. There is not yet a standard method of operationalizing self‐monitoring adherence, and the results may have differed if different criteria had been used. The weight loss intervention was only 12 weeks in duration, and lower adherence rates may have been observed over time if the programme was longer. This study was conducted using a self‐selected sample of adults who were aware of that treatment would involve self‐monitoring and use of digital tools, which may not generalize to participants with other types of sampling or methods of self‐monitoring. The results also may not generalize to self‐help or remote treatment. Finally, the prescribed frequency for weight self‐monitoring increased from weekly to daily in the final 2 weeks of the programme. This makes it difficult to interpret how adherence to weight self‐monitoring changed over time, and comparing Month 3 adherence across types of self‐monitoring also was complicated by this change in prescription.

The key finding from this study is confirmation that self‐monitoring should be considered a target‐specific behaviour rather than a unitary construct when evaluating total adherence, adherence over time, and association with weight loss and physical activity outcomes. As digital self‐monitoring tools become more commonly used among individuals attempting lifestyle modification, it is important that clinical work and research in this area consider these tools independently.

## CONFLICT OF INTEREST

The authors declared no conflict of interest.

## DATA SHARING AGREEMENT

The authors support data sharing. Data reported in this publication may be available in a deidentified format to other investigators for research purposes with the approval of the first author and the Institutional Review Board after all parent study analyses have been completed and results disseminated.

## FUNDING

National Institute of Diabetes and Digestive and Kidney Diseases R21DK112741

## TRIAL REGISTRATION

http://ClinicalTrials.gov identifier NCT03337139, https://www.clinicaltrials.gov
